# Graphitizing Non-graphitizable Carbons by Stress-induced Routes

**DOI:** 10.1038/s41598-017-16424-z

**Published:** 2017-11-29

**Authors:** Maziar Ghazinejad, Sunshine Holmberg, Oscar Pilloni, Laura Oropeza-Ramos, Marc Madou

**Affiliations:** 1 Department of Mechanical Engineering, California State University, Fresno, USA; 2 Department of Mechanical and Aerospace Engineering, University of California, Irvine, USA; 3 Programa de Maestría y Doctorado en Ingeniería, Universidad Nacional Autónoma de México, CDMX, Mexico; 4 Facultad de Ingeniería, Universidad Nacional Autónoma de México, CDMX, Mexico

## Abstract

Graphitic carbons’ unique attributes have attracted worldwide interest towards their development and application. Carbon pyrolysis is a widespread method for synthesizing carbon materials. However, our understanding of the factors that cause differences in graphitization of various pyrolyzed carbon precursors is inadequate. We demonstrate how electro-mechanical aspects of the synthesis process influence molecular alignment in a polymer precursor to enhance its graphitization. Electrohydrodynamic forces are applied via electrospinning to unwind and orient the molecular chains of a non-graphitizing carbon precursor, polyacrylonitrile. Subsequently, exerting mechanical stresses further enhances the molecular alignment of the polymer chains during the formative crosslinking phase. The stabilized polymer precursor is then pyrolyzed at 1000 °C and characterized to evaluate its graphitization. The final carbon exhibits a uniformly graphitized structure, abundant in edge planes, which translates into its electrochemical kinetics. The results highlight the significance of physical synthesis conditions in defining the structure and properties of pyrolytic carbons.

## Introduction

Our understanding of carbon, a deceptively simple element, has been challenged and evolved dramatically over the last couple of decades. The discoveries of carbon nanotubes^1^ and graphene^2^ have served to overturn our perception of carbon materials structures and properties, and broadened our vision beyond the traditional diamond and graphite allotropes. Consequently, these new carbon materials have prompted a significant volume of *de novo* experimental and theoretical research aimed at understanding their synthesis and application. A good portion of these studies is concerned with establishing correlations between the synthesis method and the resulting microstructure. However, the synthesis-structure relationships in new carbon materials are still inconclusive despite their widespread industrial use.

In this context, the nature of what makes a carbon precursor graphitizable or non-graphitizable has long eluded researchers. In the original work by Rosalind Franklin^3^, it was proposed that non-graphitizable carbons were nanoporous in nature with a tendency to form sp^3^ bonds that inhibit the alignment of graphite planes and thus graphitization, even at extremely high temperatures. However, as explained more recently by Harris *et al*.^4^, graphite layers are capable of realigning themselves at high temperatures, even within a highly nanoporous structure. Furthermore, diamond, which consists of sp^3^-hybridized carbon atoms, transforms into graphite above 1700 °C and therefore sp^3^ bonds alone cannot prevent graphitization. Harris instead proposed that stable fullerenic features between the individual graphite layers are preventing the graphitization, and generating the microstructure associated with non-graphitizing carbons^4–6^.

Several other studies have attributed the degree of graphitization in organic precursors to their physical and chemical nature, thus declaring graphitizability an intrinsic property of a precursor^3,7–11^. Kipling *et al*. linked the “non-graphitizing” quality of some precursors to the absence of an extended fusion phase in their carbonization, when heated to 300–500 °C^12^. This fusion phase allows the carbon atoms to freely rearrange themselves into more thermodynamically stable graphitic planes. Instead, “non-graphitizing” precursors undergoes crosslinking phase at those temperatures, which fix the molecular structure and impede the formation of graphitic planes. Despite this inherent limitation in “non-graphitizing” polymers, multiple studies have continued to investigate alternative strategies, such as hot drawing of polymer fibers, to induce graphitization in these organic precursors^13,14^.

Herein, we demonstrate how the synergistic effect of CNT-induced electrohydrodynamic forces and mechanical compressive treatment can overcome the lack of fusion-induced rearrangement in organic precursors, thus significantly enhance their graphitization. In particular, we present a framework by which a traditionally known “non-graphitizing” polymer, Polyacrylonitrile (PAN), may be graphitized at low pyrolysis temperature, thereby challenging the current notion of graphitizing and non-graphitizing polymers. The choice of electrospinning process allows us to use electrohydrodynamic forces, enhanced by the addition of carbon nanotubes, to unwind and orient the polymer molecular chains. The polymer was thermally stabilized under calculated mechanical stress to preserve and enhance the alignment of the precursor’s chains. A myriad of characterization methods, including transmission electron microscopy, Raman spectroscopy, ohmic resistance, and electrochemical measurements, were used to measure the degree of graphitization in pyrolyzed PAN specimens. The presented results demonstrate how the physical conditions and treatments applied during the synthesis affect molecular alignment in a polymer precursor to markedly enhance its graphitizability. By revealing unexplored stress-based mechanisms for carbon graphitization, this work suggests that graphitizability is not solely a chemical property of organic precursors.

## Results

### Unwinding PAN molecular chains

Electrohydrodynamic forces offer a powerful tool to manipulate the configuration of polymer molecules embedded in electrically charged liquids. A method of choice for applying electrohydrodynamic forces is electrospinning, where an electric filed is used to draw out polymer nanofibers from a reservoir into fabric mats^15^.

**Figure 1:**
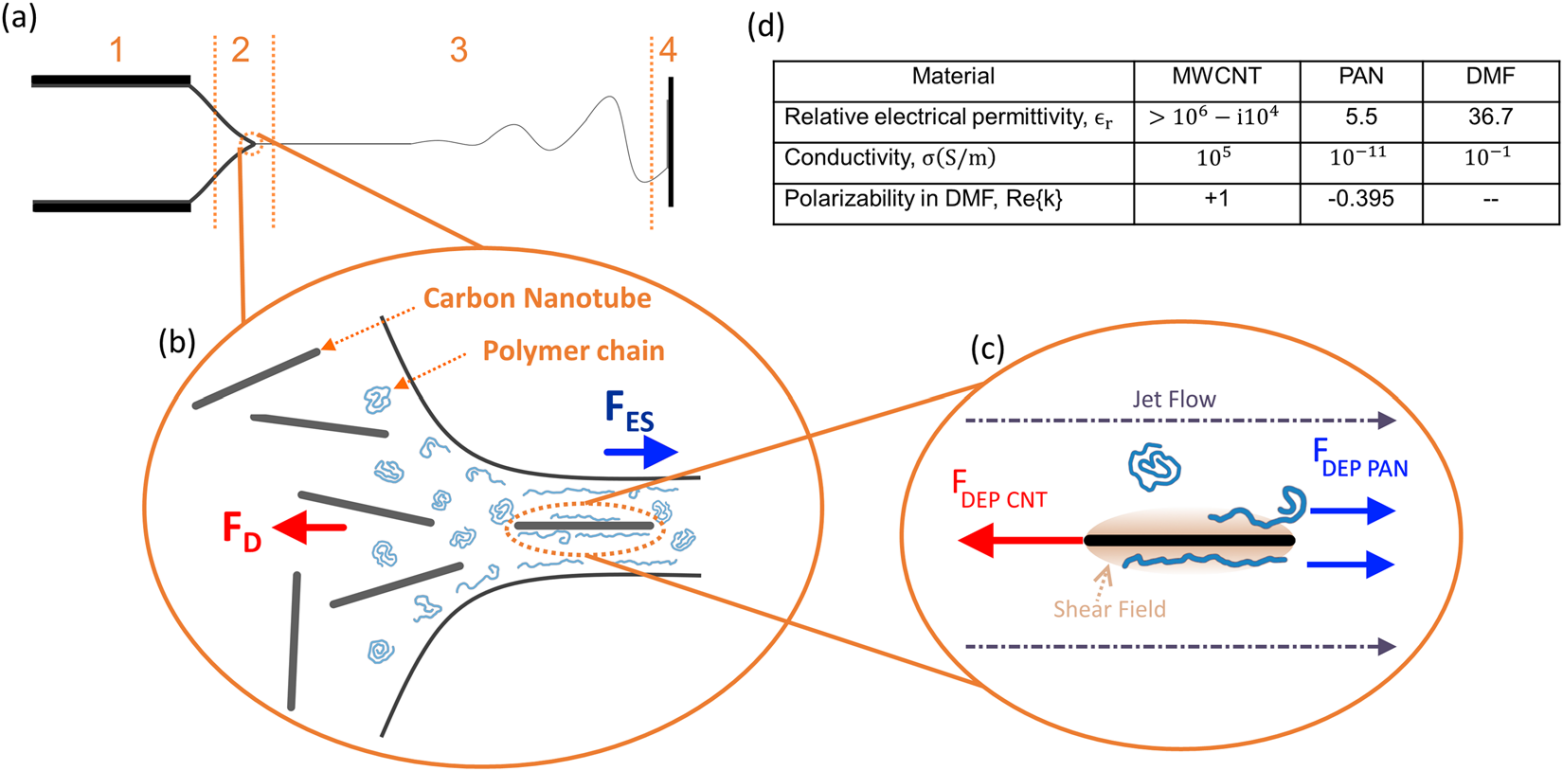
Unwinding polymer chains during PAN-CNT electrospinning process. **(a)** Different regions in the electrospinning process: (1) conducting capillary tube, (2) Taylor cone, (3) traveling jet, and (4) receiving plate. **(b)** F_ES_ is the external electrostatic force pulling the polymer jet to the receiving plate and F_D_ is the molecular drag force resisting the F_ES_. **(c)** F_DEP CNT_ is the dielectrophoretic force acting on the carbon nanotubes and F_DEP PAN_ is the dielectrophoretic force acting on the polymer chains. (**d)** Dielectrophoretic constants for multiwalled carbon nanotubes (MWCNT), polyacrylonitrile (PAN) and dimethylformamide (DMF).

The underlying mechanism driving the electrospinning process is the interplay between molecular drag forces (F_D_) in the electrospun polymer and the external electrostatic forces (F_ES_), as illustrated in Fig. 1A. The electrostatic forces deform the meniscus of the polymer fluid protruding from the capillary, which forms a region called the Taylor cone^16,17^ (Region 2 in Fig. 1). When the Taylor cone is formed, the cross-sectional area of the polymer jet reduces from that of the capillary tube to the significantly smaller cross section of the traveling jet. This change of cross sections brings about transition regions with drastically different flow velocity: a slow region within the polymer meniscus and a fast region within the traveling jet. The high velocity gradient zones developed at these interfaces result in sizable shear stress fields, which unwind the polymer chains in the jet flow^17^. Small-angle neutron scattering (SANS) and fluid mechanics studies have corroborated this confinement effect, and found a correlation between the gyration radius of the polymer, radius of the electrospun nanofiber, and the alignment of the polymer chains^18–21^.

The addition of MWCNTs into the polymer flow introduces dielectrophoresis phenomenon into the process. Here, the dielectrophoretic force (*F*_*DEP*_) stems from the difference in the electrodynamic properties of the MWCNTs, polymer (PAN) and solvent medium (DMF) in the presence of a strong non-uniform electric field (Table in Fig. 1). The direction of the *F*_*DEP*_ is determined by the dimensionless Clausius-Mossotti factor (*k*) which depends on the complex permittivity of the particle $({\varepsilon }^{\ast }=\varepsilon -\frac{\sigma }{i\omega }\,),$and the medium $\,({\varepsilon }_{m}^{\ast })$ ^22,23^:

1 $${F}_{DEP}\propto {\varepsilon }_{m}^{\ast }\mathrm{Re}\{k\}\nabla {E}^{2}$$

where,

2 $$k=\frac{{\varepsilon }_{p}^{\ast }-{\varepsilon }_{m}^{\ast }}{{\varepsilon }_{p}^{\ast }+2{\varepsilon }_{m}^{\ast }}$$

By substituting the permittivity values in Fig. 1, the *k* factor is determined to be positive for MWCNTs and negative for PAN molecular chains. Consequently, the *F*_*DEP*_ will drag the CNTs against the jet flow, while it pushes the polymer chains along the jet’s flow direction. The interaction of these opposing forces generates additional shear force zones at the surfaces of the MWCNT that further align the polymer chains.

### Formation of Stabilized PAN fibers

**Figure 2:**
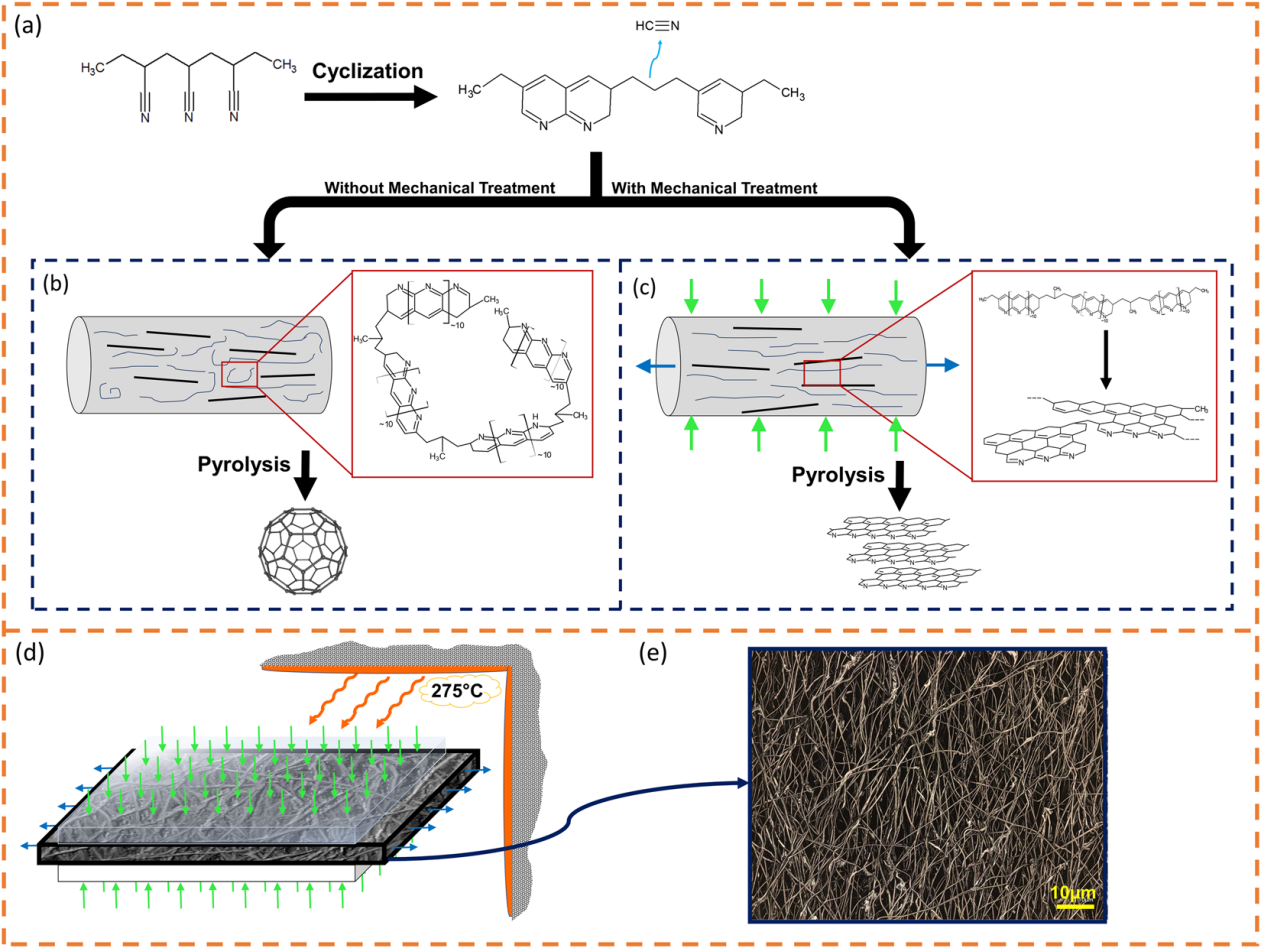
The influence of stabilization process on the microstructure of the pyrolyzed PAN fibers. **(a)** Non-ideal cyclization of PAN, which typically occurs in standard stabilization steps^28^. **(b)** Pyrolysis of as-electrospun PAN-CNT fibers (without mechanical treatment) results in amorphous carbon, and at higher temperatures in fullerenic microstructures. **(c)** Mechanical treatment of PAN-CNT fibers during the stabilization preserves the molecular alignment, which results in graphitic microstructures after pyrolysis. **(d)** Mechanical treatment of PAN fibers during the stabilization process. The electrospun PAN mat undergoes principal compressive strain and lateral tensile strain while being stabilized at 275 °C. **(e)** Confocal microscope image of the pyrolyzed PAN fibers.

A critical stabilization phase is usually carried out prior to pyrolysis by heat treatment of the polyacrylonitrile under ambient air pressure (Fig. 2)^24–27^. In the course of stabilization, PAN molecular chains gain structural stability by cross-linking with the neighboring chains, thus forming conjugated ladder-like structures. The resulting structural stability enables PAN to withstand the extreme heat treatment of the subsequent pyrolysis step^24,28^.

During the stabilization of PAN, the polymer backbone chains tend to lose nitrogen groups in the cyclization process, resulting in sp^3^ hybridized carbon bonds (Fig. 2a)^24,28^. This leads to the formation of penta- and hepta-carbon rings, which due to their curved structures will yield amorphous carbon or fullerenes during the pyrolysis. Thermodynamic stability of fullerene structures inhibits the polymer from graphitizing into carbon planes even at extremely high temperatures. In other words, the foundation of the polymer microstructures is cast during the preceding synthesis and stabilization phases. Once the curved sp^3^ carbon structures form in the stabilization, the PAN chains are set up to form fullerenes. Accordingly, high-temperature pyrolysis cannot force the underlying carbon foundation to form planar sp^2^ carbon planes, rendering the polymer “non-graphitizing”.

Considering the impact of the stabilization phase on the orientation of the PAN molecular chains, one expects that the physical parameters of stabilization would influence the graphitization of the polymer precursors substantially. The gains of polymer chain alignment from electrospinning might well be undone by the thermal stresses induced during stabilization, an insight that seems to have been overlooked. Accordingly, during the stabilization process one should aim at providing conditions that preserve or further align the PAN precursor chains, which are already oriented by the shear fields in the electrospinning process. To implement this alignment process, one can apply mechanical forces to maintain/enhance the orientation of the PAN precursor chains during stabilization (Fig. 2b).

This approach simply boils down to the question of “how to mechanically suppress the formation of curved surfaces in the polymer microstructure during the stabilization process”. It is thus anticipated that applying surface compression and/or mechanical stretching to a stabilizing polymer mat will translate into the alignment of precursor chains at a molecular-level.

Following the outlined hypothesis, we performed mechanical treatment of the polymer fibers by subjecting the PAN mats to approximately 200 kPa compressive stress while they are being stabilized at 275 °C in an oven. By performing the stabilization process under such mechanical treatment, the carbon chains are maintained partially aligned and kept from curving during this formative step. This in turn reduces the extent of fullerene formation and sets up the stabilized polymer to form more graphitic carbon planes. We also investigated the influence of applying tensile mechanical stress to the stabilizing polymers. Interestingly, changing the axial stress from compressive to tensile affects the microstructure of final carbon fibers. We discuss this effect in our upcoming report.

Subsequently, we pyrolyzed pure PAN, CNT infused PAN (PAN-CNT) and mechanically-treated PAN-CNT (M-PAN-CNT) samples at 1000 °C. The resulting carbons’ microstructure and properties were then characterized to investigate the effects of polymer chain alignment on the degree of graphitization.

### Raman Spectroscopy and Conductivity Characterization

**Figure 3:**
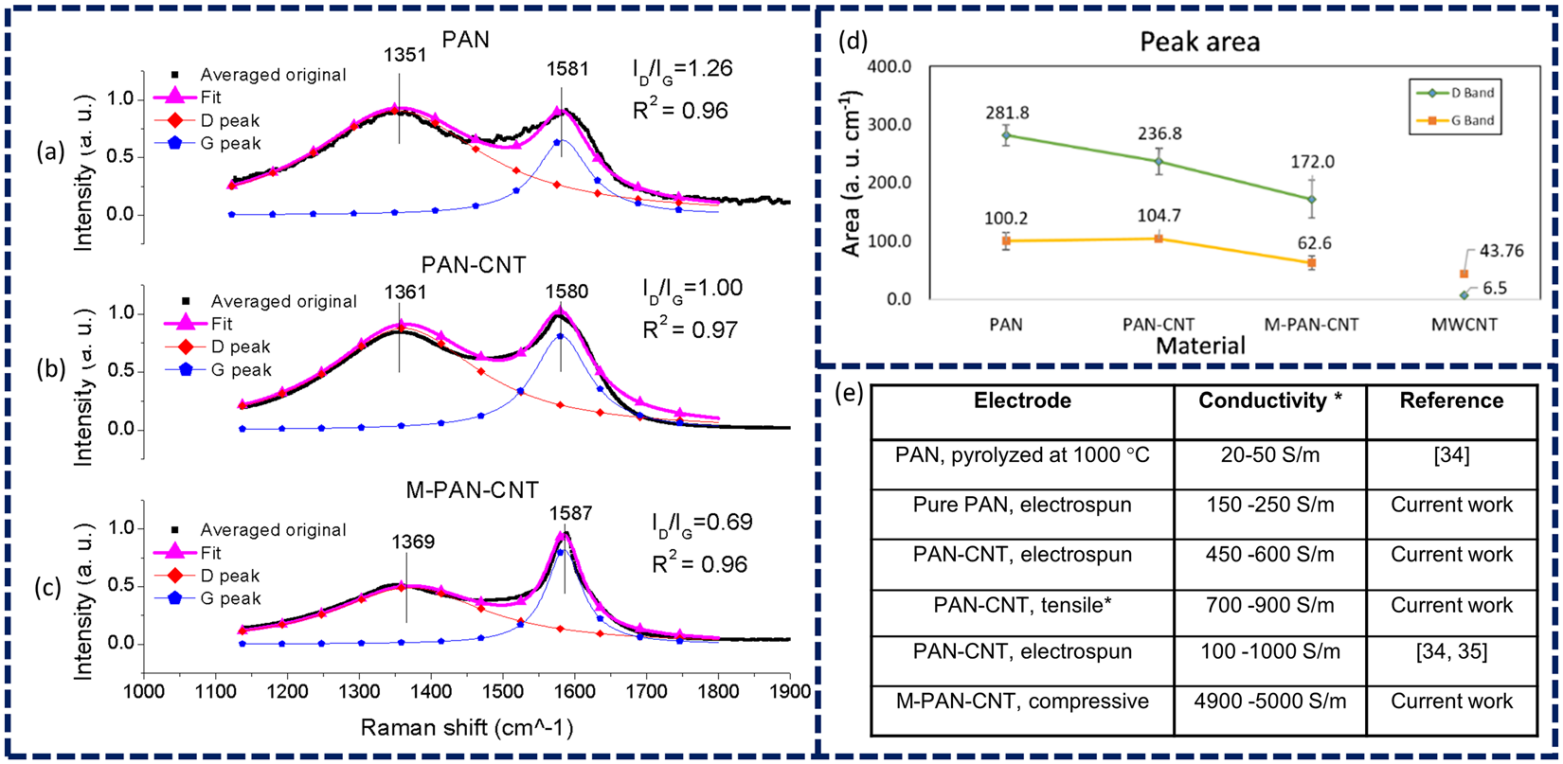
Raman spectroscopy and conductivity measurements of the PAN-based pyrolytic carbons. Raman Spectra and Lorentzian fits for pyrolyzed electrospun mats made of **(a)** PAN, **(b)** PAN-CNT and **(c)** M-PAN-CNT. Averages of over 100 collections are used to represent each type of mat. (λ _excitation_ = 532 nm) **(d)** Variation of D peak and G peak areas as functions of synthesis procedure. **(e)** Conductivity of the PAN-based pyrolytic carbon samples measured using 4-point probe method. Included for comparison, conductivities of pyrolyzed PAN carbons from other studies^54,55^. *The conductivity of the tension treated PAN-CNT carbons are measured for comparison with the compression-treated M-PAN-CNT carbons.

Over 100 Raman spectra were collected and averaged across each type of carbon fibers to characterize the microstructure of the resulting carbons. Lorentzian curves were then fitted to the D and G peaks of the representative Raman spectra for each type of synthesis (Fig. 3a–c). The heights, shapes, positions, and areas of Raman peaks can provide valuable information about structural characteristics of the final carbons^29,30^. Typically, the G peak, centered at 1560–1600 cm^−1^, is activated by vibration of carbon atoms in sp^2^-hybridized graphene planes. Thus, the height and sharpness of the G peak reveals the presence of crystalline graphitic carbons. The D peak at 1300–1400 cm^−1^ is the defect-induced band that indicates the degree of disorder in graphitic microstructures. Accordingly, the ratio of D band to G band peak heights, (*I*_*D*_)/(*I*_*G*_), has been consistently used to probe the graphitic quality of carbon materials microstructure^29–33^.

A quick examination of Lorentzian fitted Raman spectra indicates that with the application of CNTs during electrospinning, *I*_*D*_ decrease with respect to *I*_*G*_ and both peaks exhibit approximately the same intensities. The implmentation of mechanical treatment reduces the relative intensity of disorder associated band by more than 30%. Accordingly, (*I*_*D*_)/(*I*_*G*_) ratio decreases from 1.26 in pure PAN fibers to 0.69 for M-PAN-CNT fibers, revealing a remarkable increase in the graphitic structure of the resulting carbons (Fig. 3a–c).

The evolution in G and D peak areas, from PAN control samples to M-PAN-CNT fibers, supports the same upward trend of graphitization in the carbon fibers (Fig. 3d). Typically, broader Raman peaks with larger areas are correlated with a higher level of disorder and defects in the graphitic materials. The aquired Raman peak areas verify that mechanical treatment has markedly reduced the areas of D and G peaks, and thus resulted in lower level of disorder in the sp^2^ network. Interstingly, the transistion from PAN-CNT to M-PAN-CNT exhibits larger drop in the peak areas, compared to that of pure PAN to PAN-CNT. Moreover, the appreciable impact of mechanical treatment on the Raman peak areas demonstrates that the improvement of the Raman quality index (*I*_*D*_)/(*I*_*G*_) is not due to the contribution of nanotubes, as mere addition of CNTs does not change the area of the G peak.

The electrical conductivities of the PAN-based carbons, measured by four-point probe method, confirms the increasing trend of graphitization, from pyrolyzed pure PAN, to PAN-CNT, to M-PAN-CNT, with conductivity values of ~200 S/m, 550 S/m, and 5,000 S/m, respectively (Fig. 3e). The correlation between the microstructures and bulk properties reveals the uniformity of graphitization across the PAN mats, and demonstrates that the enhanced graphitic microstructures translate into improved bulk characteristics in the carbon fabrics.

### High-Resolution Transmission Electron Microscopy

**Figure 4:**
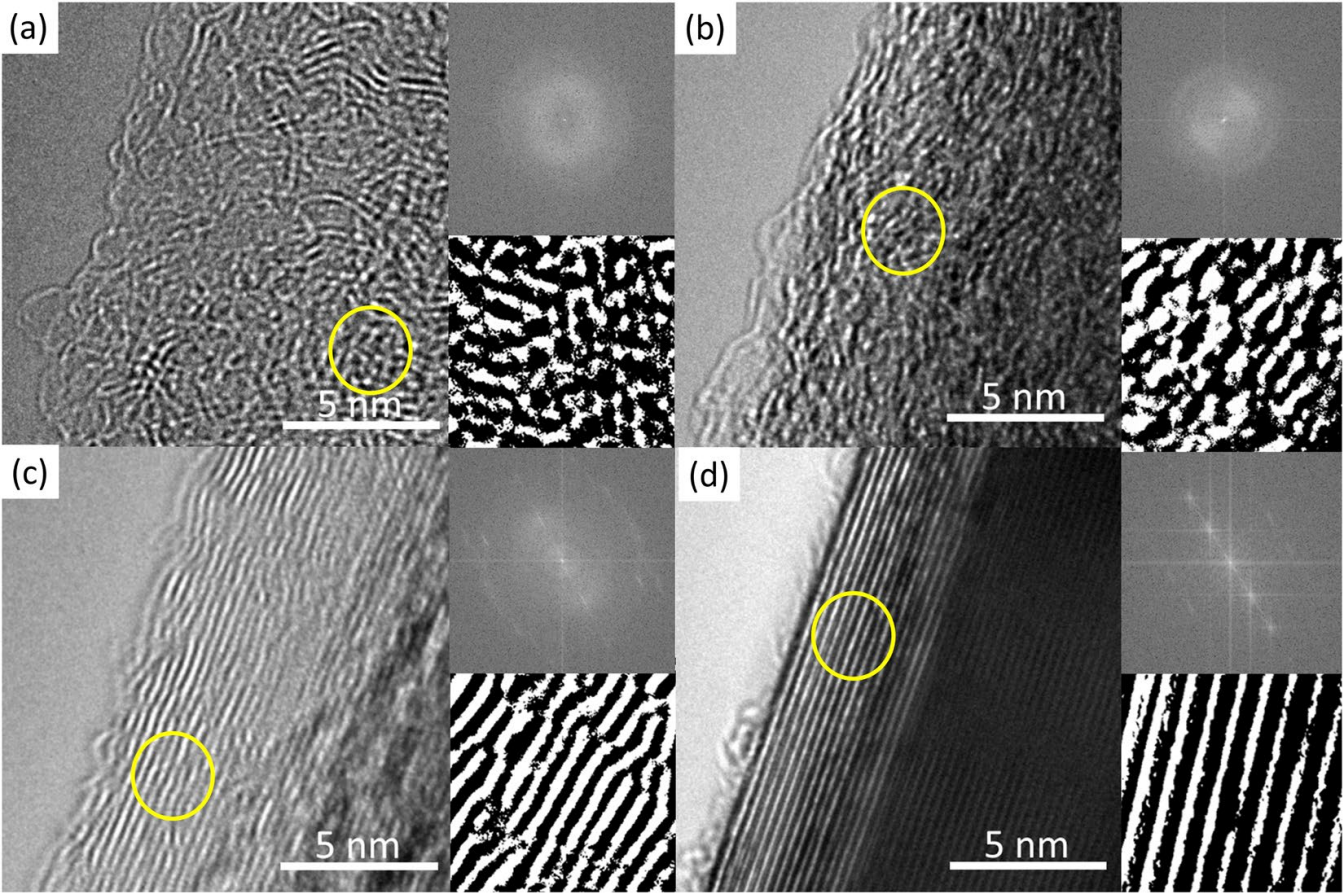
HRTEM micrographs of the PAN-based pyrolytic carbons. TEM micrographs of pyrolyzed **(a)** pure PAN, (**b)** PAN-CNT, **(c)** M-PAN-CNT and **(d)** CNT. The inset on upper right corner of each micrograph shows the associated Fast Fourier Transform of the carbon microstructures. The lower inset shows the smaller portion of the micrograph (yellow circle) enlarged and processed for enhanced visualization of the lattice fringes.

High-Resolution Transmission Electron Microscopy (HRTEM) allows for detailed study of carbon microstructure in the pyrolyzed PAN specimen. Visual examination of lattice fringes in TEM micrographs illustrates the evolution of carbon microstructures developed by the outlined processes. In Fig. 4a we show the HRTEM micrograph of the carbon fibers produced by pyrolysis of pure PAN at 1000 °C. Consistent with the agreed-upon notion that PAN is “non-graphitizing”, this micrograph reveals that the structure of the pyrolyzed PAN is highly disordered and randomly curled. The Fast Fourier Transform (FFT) of the micrograph yields symmetric rings, further evidencing the amorphous nature of the carbon fiber.

The micrographs in Fig. 4b and c reveal the effects of electrohydrodynamic molecular alignment and mechanical treatment on the structure of the resulting pyrolyzed PAN. As can be seen in Fig. 4c, the structure of M-PAN-CNT specimen exhibits aligned carbon lattice fringes, which are stacked rather tightly together. Here, the carbon lamellae are generally well-oriented with slight undulations, demonstrating imperfect graphene planes. The fast Fourier transform of this image supports our observation by revealing directionally aligned carbon planes with a fringe separation-distance of 3.6 Å.

By comparison, the nanostructure of PAN-CNT appears to be in transition from an amorphous to a more graphitic material (Fig. 4b). Here, the carbon fringes are somewhat visible but more fragmented and randomly oriented. Moreover, there is a noticeable increase in the tortuosity of the carbon planes, compared to M-PAN-CNT. In some domains, the fiber nanostructure resembles that of pure pyrolyzed PAN of Fig. 4a. This is particularly meaningful considering that randomly oriented curves in atomic planes impede the development of tightly-packed parallel carbon planes; instead, they lead to the formation of fullerenic structures^4,5,34^. Such “wrinkles” are mechanically “flattened” during the stabilization processes of M-PAN-CNT (Fig. 4c). A higher level of tortuosity also increases the fringe separation, as manifested by the 3.8 Å interlayer spacing acquired from the FFT of the TEM image (Fig. 4b inset). Figure 4d, TEM micrograph of pure MWCNT, is included for comparison with the pyrolyzed PAN samples. Expectedly, the CNT exhibits well-aligned, tightly stacked graphitic lattice planes with a minimal to zero tortuosity and fringe separation of 3.4 Å.

**Figure 5:**
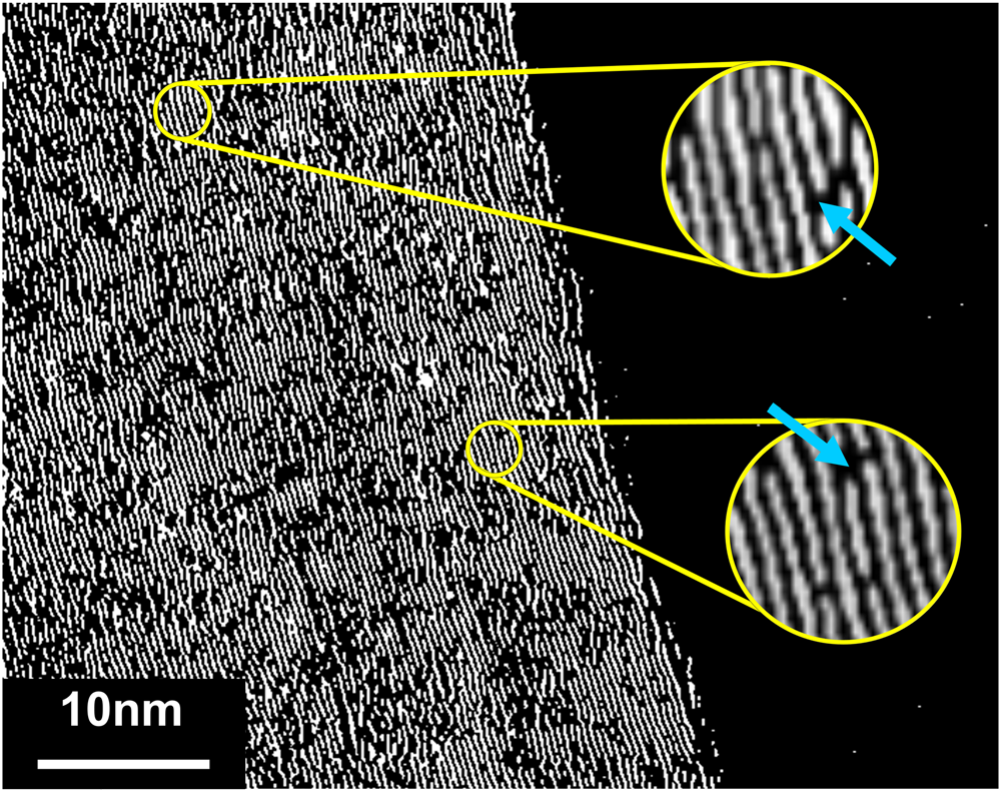
A processed image of the TEM micrograph of the M-PAN-CNT. Blue arrows point to the characteristic carbon edge planes.

To further explore synthesis-structure-function relationships in the developed carbons we resorted to image processing to visualize the carbon fringes. Such image processing allowed us to reduce the noise from the HRTEM images of the M-PAN-CNT fibers and enhance the visualization of resulting carbon microstructures. As clear from Fig. 5, the fragmented yet well-aligned lattice fringes in the M-PAN-CNT suggest a high ratio of edge planes to basal planes in the carbon structure. This observation is particularly interesting in the context of carbon’s structure-function relationships, as a number of studies attributed the electrochemical functionality of graphitic carbons to the concentration of edge plane atoms in their surface^35–37^. The proposed promise for electrochemical performance in M-PAN-CNT carbons has prompted us to evaluate the structure-functionality of the synthesized pyrolytic carbons through a comprehensive electrochemical characterization.

### Electrochemical Performance

**Figure 6:**
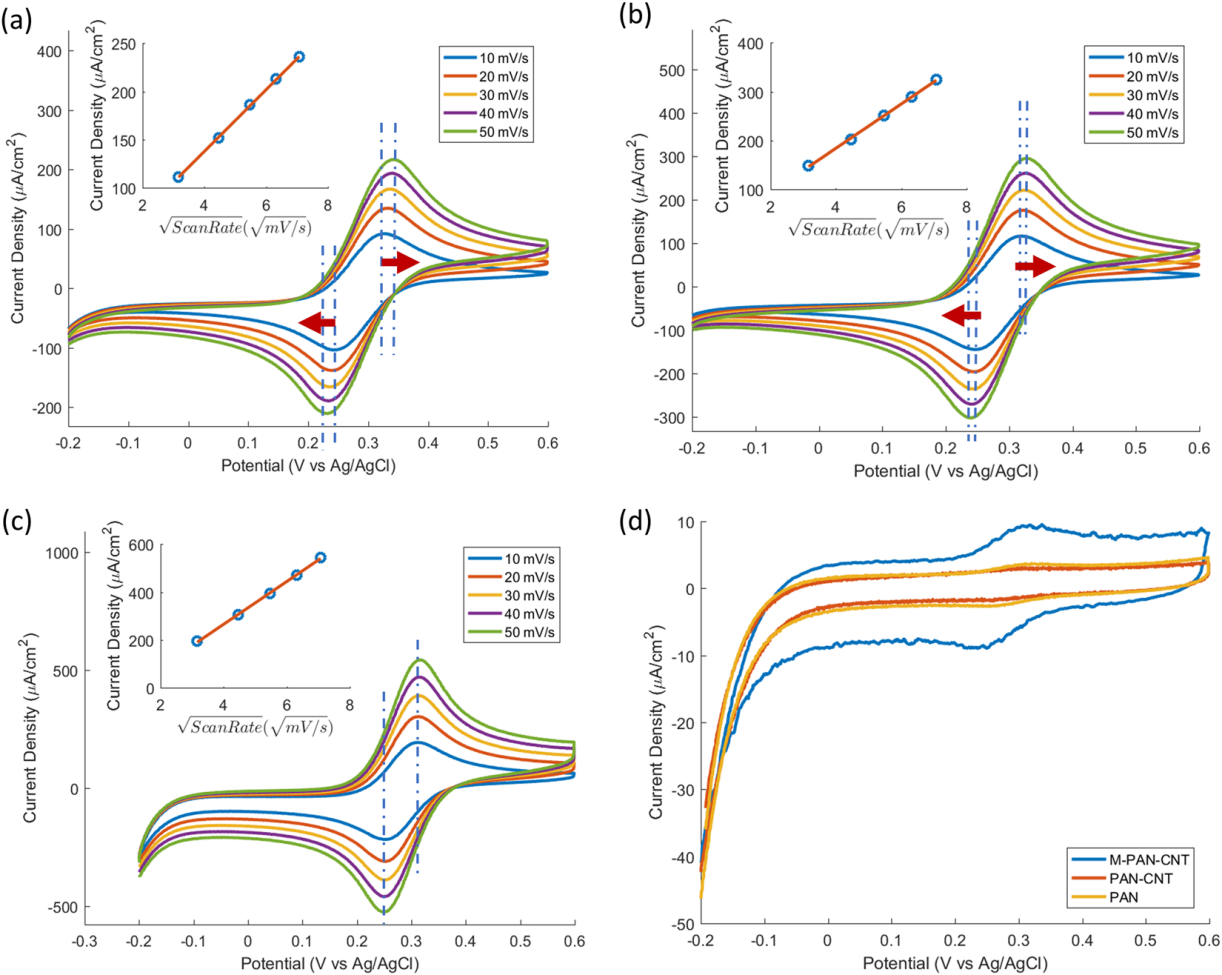
Electrochemical performance of the PAN-based pyrolytic carbons. Representative cyclic voltammograms of pyrolyzed **(a)** pure PAN, **(b)** PAN-CNT, and **(c)** M-PAN-CNT electrodes run in 1 mM potassium ferricyanide and 2 M potassium chloride aqueous solution. **(d)** The representative cyclic voltammograms of pure PAN, PAN-CNT, and M-PAN-CNT run in 2 M potassium chloride aqueous solution tested at 10 mV/s (The voltammograms were collected immediately after the [Fe(CN)_6_]^3−/4−^ characterization). All cyclic voltammograms were normalized by the geometric surface area of the pyrolyzed carbon fiber electrodes.

Cyclic voltammetry of Ferri/ferrocyanide ([Fe(CN)_6_]^3−/4−^) has been used as a standard method for evaluating the quality of a carbon electrode’s electrochemical performance^36,38,39^. The voltammograms in Fig. 6a–c allow us to evaluate the electrode’s kinetics by estimating their heterogeneous electron transfer rate constant (*k°*_*app*_) via the Nicholson method^40^,

3 $${\rm{\Psi }}({\rm{\Delta }}{E}_{peak})={(\frac{{D}_{O}}{{D}_{R}})}^{1/2}{k}_{app}^{o}{(\pi nFv{D}_{O}/RT)}^{-1/2}$$

Here, *n* is the number of electrons transferred, *F* is the Faraday’s constant, *v* is the scan rate, *R* is the universal gas constant, and *D*_*O*_ and *D*_*R*_ are oxidation and reduction diffusion coefficients for [Fe(CN)_6_]^3−/4−^, with values of 7.26 × 10^−6^ cm^2^/s and 6.67 × 10^−6^ cm^2^/s, respectively^41^. *Ψ* is a dimensionless function of *∆E*_*peak*_ (distance between the cathodic and anodic current peaks) found for the conditions of a single step, single electron transfer process at the temperature (T) of 300 K. It is important to note that the observed linear dependency between the current density of voltammograms’ peaks and the square root of the scan rate for all carbon electrodes (insets of Fig. 6) is a characteristic of standard bulk diffusion, for which Nicholson formula is applicable.

The mechanical treatment of PAN electrodes has significantly boosted their kinetics, increasing the *k°*_*app*_ eleven-fold from 0.0029 cm/s in PAN to 0.0312 cm/s in M-PAN-CNT (Table 1). The enhanced kinetics is also evident from the lack of peak shift with increasing scan rate, indicative of quasi-reversible electrochemical reaction^38^. The improvement in electrochemical performance stems from the abundance of graphitic edge planes in the carbon fiber microstructure^35,36,39^. Indeed, Compton *et al*. were able to model the electrochemical performance of carbon electrodes solely based on the quantity of graphitic edge domains on their surface^35,42,43^. Consistent with these studies, the *k* ^*o*^_*app*_ of M-PAN-CNT electrodes demonstrates an order-of-magnitude enhancement over pure CNT-based electrodes^44,45^, and approaches that of graphene and reduced graphene oxide electrodes^46,47^.

**Table 1:** Electrochemical, Raman and electrical characteristics of PAN based pyrolytic carbons compared to other carbon materials.

Carbon Type	Pyrolyzed Pure PAN	Pyrolyzed PAN-CNT	Pyrolyzed M-PAN-CNT	Glassy Carbon	MWCNT	Graphene
k^o^_app_ (cm/s)	0.0029^a^	0.0053^a^	0.0312^a^	0.0013–0.029, 0.5^b^	8.34 × 10^−5^–0.00367	1.4 × 10^−3^–0.15 ^e^
I_D_/I_G_	1.26	1.00	0.69	1.30–0.49	0.2–0.1	3–0.1^e^
σ (S/m)	150–250	450–600	4900–5000	20,000–25,000^f^	3 × 10^5 c^–2 × 10^7 d^	200–2 × 10^5, e^
Reference	Current Work	Current Work	Current Work	^36,56–58^	^44,59,60^	^44,61–63^

^a^Average k^o^_app_ measured using Nicholson method at 100 mV/s. ^b^Laser/fracturing-activated glassy carbon. ^c^Carbon nanotube yarns. ^d^Single carbon nanotube. ^e^Depends on number of defects. ^f^Pyrolyzed at 3000 °C.

All carbon fiber electrodes were tested in a 2 M KCl solution immediately after the electrochemical characterization in K_3_[Fe(CN)_6_] batch (Fig. 6d). Compared to pure PAN and PAN-CNT electrodes, the peaks in M-PAN-CNT’s voltammograms are more pronounced, pointing to stronger adsorption of [Fe(CN)_6_]^3−/4−^ onto the surface of the mechanically treated fibers. This observation is consistent with previous studies, which reported that the adsorption of [Fe(CN)_6_]^3−/4−^ onto carbon occur predominantly on functionalized graphitic edge sites^39,42,48^. It is noteworthy that the observed enhanced kinetics and adsorption capacity in M-PAN-CNT are achieved without any additional activation process. The inherent aptitude of M-PAN-CNT for functionalization is particularly attractive because it addresses a main challenge in utilizing graphitic carbons for various electrochemical devices^49–53^.

## Discussion

In this work, we discussed how electro-mechanical parameters of synthesis influence molecular alignment in a polymer precursor to enhance its graphitization. We focused on carbonization of polyacrylonitrile as the carbon precursor, since it is generally considered a “non-graphitizing polymer”, and is a main source for commercial carbon fibers. The application of electrospinning allowed us to utilize electrohydrodynamic forces, boosted by addition of carbon nanotubes, to align polymer molecular chains within the spun fibers. The polymer chain alignment was then preserved by mechanical compression of the polymer fabrics during the formative stabilization step. Subsequently, a myriad of material analysis techniques, including Raman spectroscopy, transmission electron microscopy, electrical conductivity, and electrochemical measurements allowed us to evaluate the graphitization level of the pyrolyzed PAN specimen. Pyrolyzing the curved polymer chains lead to formation of amorphous carbon or fullerenic structures, whereas mechanically aligned carbon precursors are poised to form more graphitic carbon planes. This result underlines the correlation between the physical conditions exerted on a stabilizing polymer and its graphitization, thereby, emphasizing that the foundation of the final carbon structure is cast during the stabilization of the precursor polymer.

Graphitization of organic precursors is a complex phenomenon and it is unlikely to find a comprehensive solution to develop graphitic carbons from different polymer precursors. This work addresses the physical aspect of polymer graphitization and explores the influence of the synthesis parameters on the microstructure of the pyrolytic carbons. By investigating the effects of applied electro-mechanical stresses during the carbon synthesis process, the presented results reveal further insight into the underlying mechanism of graphitization and provides an attractive pathway for tailoring the microstructure of carbon materials.

## Materials and Methods

### Carbon Synthesis

#### Electrospinning

For electrospinning PAN fibers, polyacrylonitrile (Sigma Aldrich) with molecular weight M_w_ = 150,000 g/mol was dissolved in DMF, N, N-dimethylformamide (Sigma Aldrich), at 40 °C for 24 hours to a final concentration of 8 wt.%. The solution for electrospinning CNT-infused PAN (PAN-CNT) nanofibers was prepared by dispersing MWCNT, multi-walled carbon nanotubes (Sigma Aldrich) with a diameter of 110–170 nm and a length of 5–9 µm, in DMF under ultra-sonication for 1 hour then stirred for 24 hours at 40 °C. Subsequently, PAN was dissolved into the DMF-CNT solution by stirring at 40 °C for 24 hours, to achieve a final concentration of 8 wt.% PAN and 1 wt.% MWCNT. Electrospinning for PAN and PAN-CNT solution was performed by loading the PAN or PAN-CNT solutions, respectively, into a 1 mL syringe mounted on a syringe pump, which provided a flow rate of 0.7 mL/hour. An operating voltage of 15 kV was applied between a 21-gauge needle (820 µm outer diameter) and an 8 × 8 cm^2^ aluminum plate, which was held at 15 cm away from the dispensing needle. The electrospinning process was run for 1 hour to form a ~100 µm thick PAN and PAN-CNT nanofiber mats, respectively.

#### Stabilization

PAN and PAN-CNT mats were peeled off the aluminum plate and then thermally stabilized on top of an alumina plate at 275 °C in an oven for 5 hours. For mechanically-treated PAN-CNT (M-PAN-CNT) samples, a PAN-CNT mat was subjected to a 200 kPa compressive stress between two alumina plates, while being stabilized in an oven at 275 °C for 5 hours.

#### Pyrolysis

All stabilized PAN nanofibers were pyrolyzed in a tube furnace under continuous flow of Nitrogen at an approximate flow rate of 4,600 sccm. First, the stabilized samples were heated to 300 °C at a 4.5 °C/min ramp rate and maintained at that temperature for one hour. Next, the temperature was increased to 1000 °C at a 2.5 °C/min ramp rate and held at that temperature for an hour before cooling down to ambient temperature at 10 °C/min rate.

### Characterization

The pyrolyzed polymer samples characterized by Raman spectroscopy with a Renishaw InVia Raman Microscope, equipped with 532-nm excitation laser set, to assess the graphitization of the pyrolyzed PAN samples. Over 100 Raman spectra were acquired and averaged across each type of carbon fibers to evaluate the graphitic quality of the resulting carbons. The Raman spectra were then fitted using Lorentzian curves and analyzed. Optical confocal microscopy (Keyance, VK-X) and scanning electron microscopy (FEI, Quanta 3D FEG Dual Beam) were employed to observe the morphology of the carbon nanofibers. 4-point probe station (Signatone, S-302-4, with a SP4 probe head) was used to measure the conductivity of the pyrolyzed PAN samples.

High-resolution transmission electron microscopy (HRTEM; FEI, Titan 80–300 kV S/TEM) operated at 300 kV was used to visualize and evaluate the microstructure of the pyrolyzed PAN samples. TEM samples were prepared by drop-casting pyrolytic carbon sample, suspended in toluene, onto TEM grids (PELCO CM20 purchased from Ted Pella, Inc.). Fast Fourier Transform (FFT) was applied on the collocated TEM micrographs to further measure the crystallinity and the fringe separation distance of the resulting carbons.

Cyclic voltammetry (CV) was used to characterize the electrochemical behavior of the three distinct types of carbon fiber mats(CFM): pure PAN, PAN-CNT and M-PAN-CNT. CVs of the CFM were first run in a 1 mM potassium ferricyanide (Fischer Scientific) in 2 M potassium chloride (Sigma Aldrich) solution using a traditional 3-electrode configuration with a Ag/AgCl (BASi) reference electrode and a 1 cm^2^ polished glassy carbon (Structure Probe, Inc) counter electrode. Potassium ferricyanide was chosen as the redox couple to characterize the CFM as it is particularly sensitive to the presence of graphitic edge planes, while remaining relatively insensitive to most other surface modifications^36,38^. To further distinguish the effects of graphitic edge planes, a subsequent CV is run in a 2 M KCl aqueous solution. The presence of redox peaks in this second CV affirms the presence of ferricyanide ion adsorption along the edge planes^35,36^. To characterize the electrochemical performance of the pyrolyzed PAN electrodes, the heterogeneous electron transfer rate, *k* ^*o*^_*app*_, was calculated using the Nicholson method^40^,

3 $${\rm{\Psi }}({\rm{\Delta }}{E}_{peak})={(\frac{{D}_{O}}{{D}_{R}})}^{1/2}{k}_{app}^{o}{(\pi nFv{D}_{O}/RT)}^{-1/2}$$

In equation (3), *n* is the number of electrons transferred, *F* is the Faraday’s constant, *v* is the scan rate, *R* is the universal gas constant, and *D*_*O*_ and *D*_*R*_ are oxidation and reduction diffusion coefficients for Ferricyanide redox couple, with values of 7.26 × 10^−6^ cm^2^/s and 6.67 × 10^−6^ cm^2^/s, respectively^41^. *Ψ* is a dimensionless function of *∆E*_*peak*_ (distance between the cathodic and anodic current peaks) found for the conditions of a single step, single electron transfer process at the temperature (T) of 300 K. *k* ^*o*^_*app*_ is determined by evaluating the *∆E*_*peak*_ at a scan rate of 100 mV/s of each pyrolyzed PAN nanofiber mats.

## Electronic supplementary material

Supplementary Information
