# Supplementary material for: Graphitizing Non-graphitizable Carbons by Stress-induced Routes
Source: Sci Rep. 2017 Nov 29;7:16551. doi: 10.1038/s41598-017-16424-z (PMC5707352; doi:10.1038/s41598-017-16424-z)
Supplement: Supplementary file 1 — Supplementary Information [file 41598_2017_16424_MOESM1_ESM.pdf]

Supplementary Materials for

Graphitizing Non-graphitizable Carbons by Stress-induced Routes

Maziar Ghazinejad[1,2], Sunshine Holmber[1], Oscar Pilloni[3], Laura Oropeza-Ramos[4] and Marc Madou[1*]

[1]Department of Mechanical and Aerospace Engineering, University of California, Irvine

[2]Department of Mechanical Engineering, California State University, Fresno

[3] Programa de Maestría y Doctorado en Ingeniería, Universidad Nacional Autónoma de México, México

[4] Facultad de Ingeniería, Universidad Nacional Autónoma de México, México

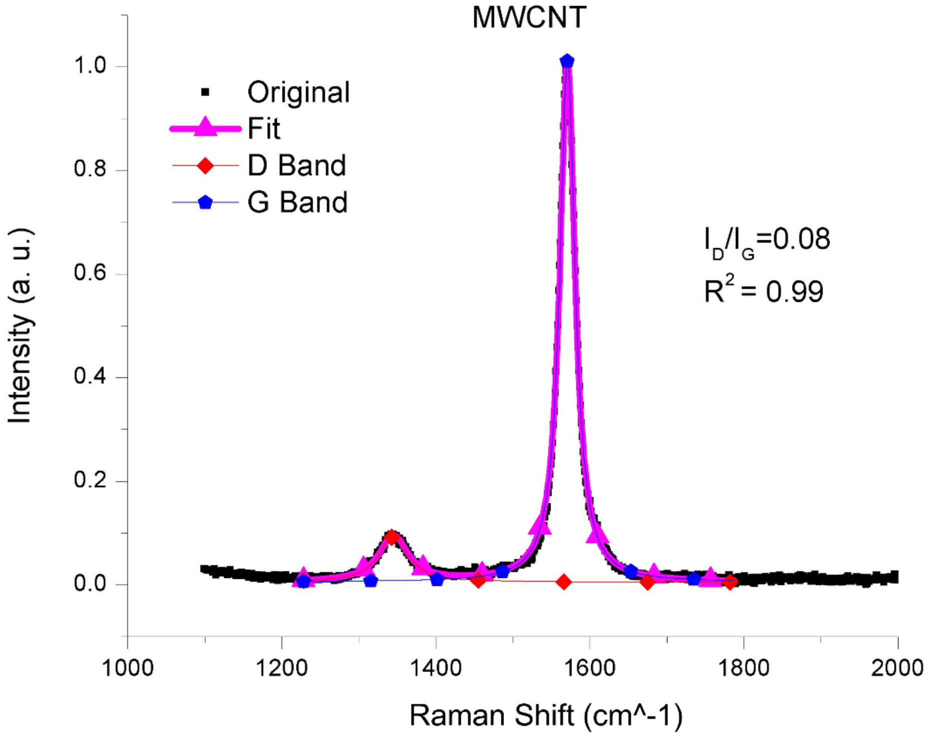

Figure S1. Raman spectrum and Lorentzian fits of the carbon nanotubes used in the electrospun polymer nanofibers. $\lambda_{excitation}$ = 532 nm
